# Cranial neural tube defect after trimethoprim exposure

**DOI:** 10.1186/s13104-018-3593-1

**Published:** 2018-07-16

**Authors:** Nor Linda Abdullah, Renuka Gunasekaran, Siti Waheeda Mohd-Zin, Bee-Hui Lim, Pramila Maniam, Anis Shuhada Mohd-Salleh, Meow-Keong Thong, Zamri Chik, Noreena Nordin, Zaliha Omar, Julia Patrick Engkasan, Dharmendra Ganesan, Zakaria Nurul Aiezzah, Azlina Ahmad-Annuar, Noraishah Mydin Abdul-Aziz

**Affiliations:** 1 Department of Parasitology, Faculty of Medicine, University of Malaya, 50603 Kuala Lumpur, Malaysia; 2 Molecular Pathology Unit, Cancer Research Centre, Institute for Medical Research, Jalan Pahang, 50588 Kuala Lumpur, Malaysia; 3 Perdana University Graduate School of Medicine, Block B and D1, MAEPS Building, MARDI Complex, Jalan MAEPS, 43400 Serdang, Selangor, Malaysia; 4 Department of Paediatrics, Faculty of Medicine, University of Malaya, 50603 Kuala Lumpur, Malaysia; 5 Department of Pharmacology, Faculty of Medicine, University of Malaya, 50603 Kuala Lumpur, Malaysia; 6 Department of Pharmacology, Faculty of Medicine, University of Malaya Bioequivalence and Testing Centre (UBAT), University of Malaya, 50603 Kuala Lumpur, Malaysia; 7 Department of Rehabilitation Medicine, Faculty of Medicine, University of Malaya, 50603 Kuala Lumpur, Malaysia; 8 Department of Surgery, Faculty of Medicine, University of Malaya, 50603 Kuala Lumpur, Malaysia; 9 Unit of Pathology & Transfusion, Hospital Parit Buntar, Jalan Sempadan, 34200 Parit Buntar, Perak, Malaysia; 10 Department of Biomedical Science, Faculty of Medicine, University of Malaya, 50603 Kuala Lumpur, Malaysia

**Keywords:** Neural tube defects, Trimethoprim, Folic acid antagonist, Acne, Primary neurulation, Malaysia

## Abstract

**Objectives:**

The Neural Tube Defects Research Group of University of Malaya was approached to analyze a tablet named TELSE, which may have resulted in a baby born with central nervous system malformation at the University of Malaya Medical Centre. In this animal experimental study, we investigated the content of TELSE and exposure of its contents that resulted in failure of primary neurulation.

**Results:**

Liquid Chromatography Tandem Mass spectrophotometry analysis of the TELSE tablet confirmed the presence of trimethoprim as the active compound. The TELSE tablet-treated females produced significant numbers of embryos with exencephaly (n = 8, 36.4%, *P < 0.0001), in all litters. The TELSE tablet-treated females subsequently given folic acid did not result in pregnancies despite there being evidence of possible resorption. Furthermore, after multiple rounds of mating which did not yield viable pregnancies, eventually, 2 embryos with exencephaly were harvested in a litter of 6 at 0.05% w/v pure trimethoprim once. The use of trimethoprim, a folic acid antagonist, peri-conceptionally increased the risk of exencephaly in the mouse.

**Electronic supplementary material:**

The online version of this article (10.1186/s13104-018-3593-1) contains supplementary material, which is available to authorized users.

## Introduction

Spina bifida and anencephaly, the two most common forms of neural tube defects (NTDs), occur in 1–10 per 1000 births worldwide [1]. The prevalence of NTDs in Malaysia is high; in University of Malaya Medical Centre alone, as many as 2–9 cases of spina bifida is reported in a cohort of a thousand yearly [2]. NTDs are a result of complex interaction between genetic and environmental factors [3, 4]. Several known environmental risk factors for NTDs include maternal diabetes [5], certain antiepileptic drugs such as valproic acid and phenytoin [6, 7], folic acid antagonists [8, 9], hyperthermia during early pregnancy [10], poor nutrition [11], and low socioeconomic status [1, 3]. Multiple candidate genes have been found to be associated with NTDs [1, 12]. For example, NTDs have been linked to several genes in the folate-homocysteine metabolic pathway, consistent with epidemiological evidence that between 30 and 70% of NTDs can be prevented by prenatal folate [13]. Extensive research has shown that peri-conceptional folic acid supplementation can markedly reduce about 70% of the re-occurrence risk of NTD-affected pregnancies [14, 15]. Therefore, it is recommended that a daily supplement of folic acid for women planning their pregnancy and during pregnancy to be that of 0.4 mg [3, 16, 17]. However, a higher dose of 4 mg should be given to those with pre-existing diabetes, obesity, and where there is previous delivery of infant with NTDs or family history of NTDs [18].

In this study, we report the first case of a tablet-suspected trimethoprim, named TELSE, so named by the dermatologist who prescribed the drug to a woman at a dermatology clinic for a duration of approximately 4 months that may have resulted in the birth of her child with a central nervous system malformation. The woman was prescribed TELSE (which we later found out to contain trimethoprim) and upon discovering she was pregnant, sought advice from the clinic which prescribed her the medication whereby she was then told to discontinue the medication. The mother then proceeded to consume folic acid throughout her pregnancy. We wanted to understand if this was the possible reason as to why the child had survived post-natally as she was born with a skin-covered cyst where her brain was supposed to have been. Trimethoprim is a folic acid antagonist and the use of folic acid antagonist peri-conceptionally has also been associated with neural tube defects [8, 9]. We report the analysis of the content of the tablet and carried out the pharmacological exposure during primary neurulation and as well as attempted rescue with folic acid in an animal study.

## Main text

### Methods

#### Animal study

Specified pathogen free outbred CD1 mice were supplied from BioLasco, Taiwan Co., Ltd and maintained in an Animal Biosafety Level-2 facility (Malaysia). Outbred CD1 female mice aged 6-weeks old were divided into five experimental groups of two mice each (Additional file 1). Total ten outbred CD1 female mice were used in this experiment and they weighed between 23–25 and 26–27 g before and after mating at E10.5, respectively. Group One was given 0.5% w/v TELSE tablet dissolved in drinking water. Each tablet weighed 0.5 g, which amounts to 100 mg of trimethoprim (as determined by Liquid Chromatography Tandem Mass Spectrometric (LC-MS/MS), which was subsequently dissolved in 100 ml of water [19]. The TELSE-infused drinking water at 0.5% w/v, which contained 0.1% w/v trimethoprim, was provided ad libitum to the female mice in Group One. The TELSE-infused water was the sole source of drinking fluid in Group One. Group Two was only given water ad libitum. The average liquid consumption was monitored. After 1 month of treatment, female mice were placed with a healthy CD1 male stud for overnight mating. Females with copulation plug were designated as embryonic day 0.5 (E0.5). Somite numbers and crown-rump length were counted as a measure of developmental progression.

Group Three was exposed to TELSE as described above in Group One and subsequently given folic acid (0.0004% w/v) (Iberet ^®^ Folic 500, Abbott, USA) the day after copulation until the targeted E10.5 embryo harvest. On the day that folic acid treatment began, the TELSE-infused drinking water was stopped. At early E10.5 (between 22 and 26 somites stage), the pregnant female mice were euthanized by cervical dislocation and embryos were dissected in Dulbecco’s modified Eagle’s medium (Nacalai Tesque, JAPAN) containing 10% fetal calf serum (Sigma, USA).

An additional study using pure trimethoprim (GoldBio^®^) were performed at the concentrations of 0.1% w/v (Group Four) and 0.05% w/v (Group Five). Five rounds of mating were repeated at each dose in order to yield embryos for assessment. Embryos were assessed using a high-resolution stereomicroscope (Leica MZ16, GERMANY). Embryo dissection and scoring were blinded and randomised. The respective treatment was revealed only during image capture and analysis.

#### Statistical analysis

Statistical analysis was carried out using GraphPad Prism 5.0 (GraphPad Inc., USA). Comparisons of mean values were made by two-way Analysis of Variances (ANOVAs). Subsequently, P < 0.001 was designated as statistically significant.

### Liquid Chromatography Tandem Mass spectrophotometry (LC-MS/MS) analysis

#### Identification of trimethoprim

Identification and quantitation of trimethoprim in the tablets obtained from dermatological clinic which prescribed medication for acne was carried out by LC-MS/MS system (ABSciex, API 3200). 1 mg of crushed tablet was dissolved in 100% methanol and filtered using 0.45 µm-syringe filter. A 10 µL solution was injected into LC-MS/MS system. The analytical columns used were Phenomenex, Gemini-NX C18 (150 mm length × 2.1 mm I.D, particle size 5 µm) and Phenomenex, Gemini-NX C18 (4 mm ID × 2.0 mm length). The mobile phase used was 0.1% formic acid and 100% acetonitrile, respectively. The flow rate was set at 0.30 mL/min involving gradient elution at room temperature. Trimethoprim transition ions were monitored in the multiple reaction monitoring (MRM) at *m/z* 291 and *m/z* 230.

#### Determination of Amount of Trimethoprim in tablets

Using the above method, amount of trimethoprim in each tablet was further determined by using the calibration curve of trimethoprim standard prepared in methanol covering the concentration range of 20–1000 ng/mL.

## Results

### Animal study

#### Trimethoprim treatment from tablet obtained from dermatological clinic

**Fig. 1:**
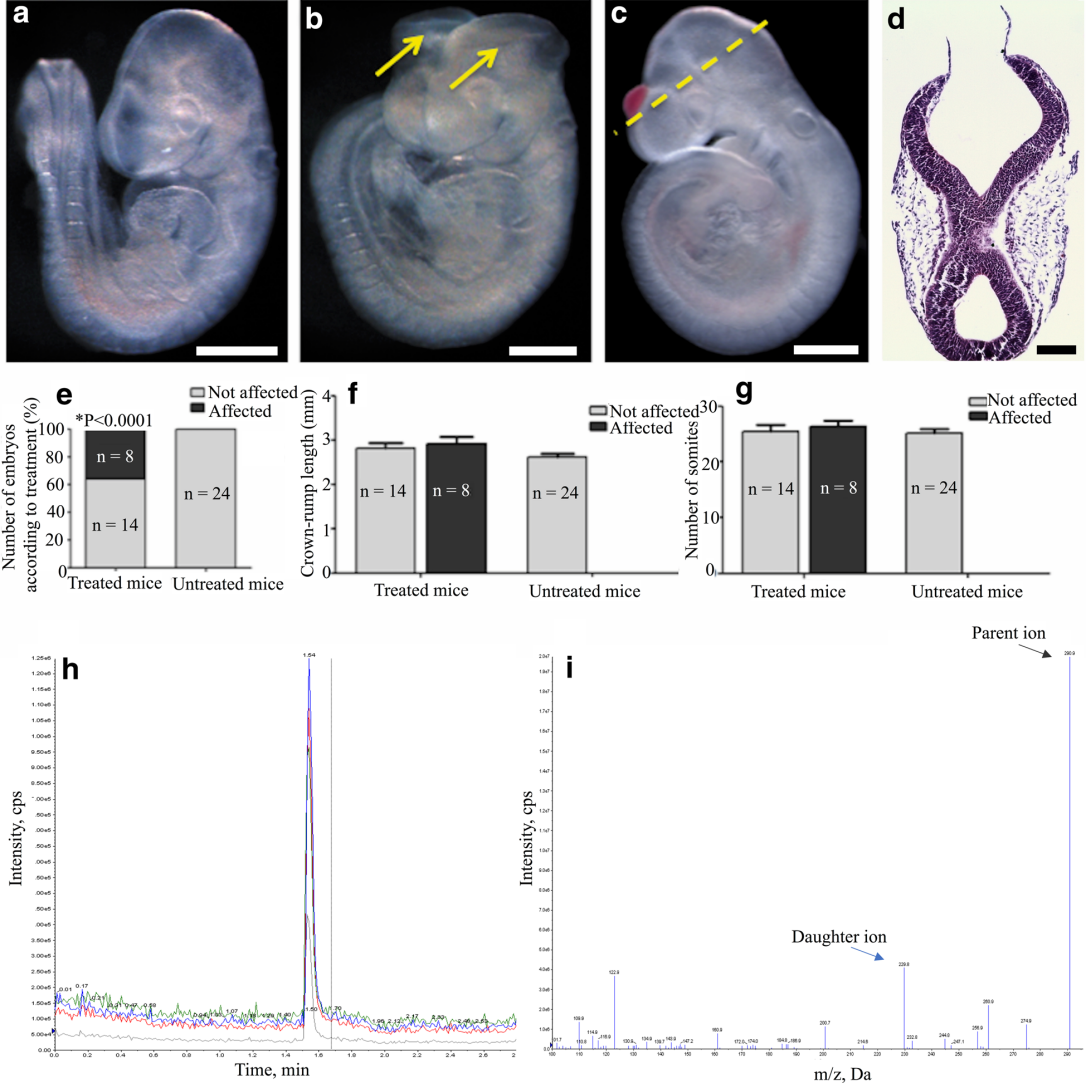
Mouse model exhibiting cranial neural tube defect after trimethoprim exposure. **a** Control mouse embryo (untreated) at E10.5 (24 somites stage) age matched against **b** mouse embryo with exencephaly at E10.5 (23 somites stage) harvested from TELSE tablet (0.5% w/v containing 0.1% w/v trimethoprim) administered female CD1 mouse. The arrows indicate the open cranial neural tube. **c** Mouse embryo with cranial neural tube defect at E10.5 (22 somites stage) harvested from pure trimethoprim (containing 0.05% w/v trimethoprim) administered female CD1 mouse. **d** Transverse section through the cranial region of embryo C showing open hindbrain (Scale bar for A-C represent 0.5 mm and D represent 0.1 mm). **e** TELSE tablet administered female CD1 mouse showed significantly increased number of exencephalic embryos (n = 8) compared to control embryos (n = 24) (*P < 0.0001) (ANOVA). **f**, **g** No significant differences were seen between crown-rump length and number of somites in the E10.5 embryos between affected (n = 8) and not affected embryos of both treated mice (n = 14) and untreated mice (n = 24) (P < 0.0001) (ANOVA) (Value of bars are medians ± standard deviation). **h**, **i** Chromatogram of trimethoprim showed a single peak at 1.54 min and mass spectrometry analysis of the TELSE tablet confirmed the presence of trimethoprim as the active compound, with an exact mass of 291 and 230 for parent and daughter ions respectively

At E10.5 (22–26 somites stage), there was a significantly higher number of embryos exhibiting exencephaly (36.4%, n = 8) produced by trimethoprim-treated females (Group One) than that of the control group (Group Two) (n = 24) (*P < 0.0001) (ANOVA) (Fig. 1a, b, e). However, there was no significant difference in the growth and development of both affected and non-affected E10.5 embryos when compared to untreated embryos (n = 24) as reflected by the crown-rump length and number of somites (Fig. 1f, g).

#### Pure trimethoprim (GoldBio^®^) treatment

Only one litter was successfully obtained from the 0.05% w/v pure trimethoprim treatment. The litter contained 6 embryos harvested at early E10.5. Two embryos exhibited an abnormal cranial region each (Fig. 1c), which were subsequently sectioned and showed an open hindbrain (Fig. 1d). The other four embryos appeared unaffected by gross abnormalities; however, this particular litter was fragile during dissection and tissues tend to disintegrate; therefore experience on the part of the scientist was required.

#### Rescue experiments with folic acid

None of the females were pregnant despite the presence of vaginal plugs. Upon dissection, the mouse uterine horns were found to be engorged but without viable embryos despite blood clots which suggests possible resorption (Additional file 2).

#### LC-MS/MS analysis

Analysis confirmed the presence of trimethoprim as the main active compound in the tablet prescribed to the patient’s mother. Chromatogram showed a single peak at 1.54 min with an exact mass of 291 *m/z* and 230 *m/z* for parent and daughter ions, respectively (Fig. 1h, i). The average amount of trimethoprim in a single TELSE tablet was approximately 100 mg. Duplicates were performed.

## Discussion

The antibiotic trimethoprim is a folic acid antagonist, which is frequently used in combination with sulfamethoxazole to treat urinary tract infections in women. Trimethoprim acts as an inhibitor of dihydrofolate reductase (DHFR) by displacing folic acid from the enzyme and thereby blocking the conversion of dihydrofolic acid to tetrahydrofolic acid. Tetrahydrofolic acid is the active metabolite in the human body, which plays a central role in numerous cellular processes, including production of purines, and thymidylate that are essential for DNA synthesis [20].

The potential risk of birth defects has been reported with the use of trimethoprim-sulfamethoxazole (TMP-SMZ) since decades ago, but the potential reproductive adverse effects associated with trimethoprim alone have only been recognized recently [21]. According to the US Food and Drug Administration (FDA), prior to 2015, trimethoprim has been assigned as a pregnancy category C drug that should be used with caution if only potential benefits outweigh potential risks to the fetus [22]. At the present time, trimethoprim has been given a new FDA established pharmacologic class (EPC) text phrase of dihydrofolate reductase inhibitor antibacterial under the Pregnancy and Lactation Labeling Rule (PLLR) [23].

### Trimethoprim is teratogenic

The use of folic acid antagonists peri-conceptionally has been associated with certain birth defects including neural tube defects, cardiovascular defects, oral cleft, urogenital malformations, and limb defects [8, 9, 21]. Folic acid antagonists such as aminopterin, methotrexate, and valproic acid are known to increase the risk of NTDs [8]. There exists few animals studies on the effect of trimethoprim on pregnant mice although a single study have shown that exencephaly increases when induced by both valproic acid and trimethoprim [24]. However, the association between trimethoprim and NTDs has only recently been established [8, 9, 21]. The toxicity of pure trimethoprim in this study was not established as we were unable to produce sufficient pregnancies in order to reach a finding. However, the toxicity of TELSE which contains trimethoprim was duly established during neurulation.

### Trimethoprim causes open cranial neural tube defects (exencephaly) in mice

Based on our animal study using the CD1 mouse as a model; affected embryos with exencephaly were significantly detected when harvested from pregnant females given trimethoprim for 1 month prior to conception and continued until embryos were harvested at E10.5 (Fig. 1b). The number of affected embryos were significant (Fig. 1e) and observed in both treated females in Group One showing the possible consistency and relation between trimethoprim and primary neurulation. Mice undergo the process of neural tube closure (neurulation) between E8.5 until E10.5. Despite the open neural tube defect in a significant number of embryos (*P < 0.0001) (Fig. 1e), the growth and development of the embryos were not affected (Fig. 1f, g) which suggests that the effect of trimethoprim is specific to neurulation. We would also suggest repeating the study by increasing the number of pregnant dams to further determine the effect of trimethoprim during primary neurulation. Nevertheless, our case report further highlights the potentially serious teratogenic effect of trimethoprim.

## Conclusion

Trimethoprim should be avoided during the first trimester of pregnancy or in childbearing women due to the potentially serious teratogenic effects. We suggest for physicians to be aware of the potential teratogenic effects of trimethoprim and should not prescribe the drug to pregnant women or those who potentially can become pregnant. If the use of trimethoprim is inevitable, we recommend physicians to fully inform the patients regarding its potential teratogenic effects. Contraceptive measures should be used during treatment with trimethoprim and for at least 3 months after treatment. Further analysis to the re-classification of trimethoprim into FDA pregnancy category D is suggested as well as the added PLLR label.

## Limitations

1. TELSE tablet used in this experiment were limited and provided by individuals given the same prescription at the same dermatology clinic by the same doctor.
2. Due to non-viable pregnancies in rescue experiments with folic acid and in the pure trimethoprim treatment, multiple rounds of mating were required. Eventually, only a lower dose of pure trimethoprim yielded a single pregnancy as mentioned in the Results Sect. 
3. In rescue experiments with folic acid, none of the females were pregnant despite the presence of vaginal plugs. However, upon dissection, the engorged uterine horns with blood clots suggested possible resorption, thus Additional file 2 was included.

## Additional files

**Additional file 1.** Schematic for animal study to look for effect of trimethoprim on primary neurulation E10.5.

**Additional file 2.** Unsuccessful pregnancy showing engorged mouse uterine horn. Despite repeated attempts at mating and with the presence of vaginal plugs, upon dissection, the engorged uterine horn with blood clots were instead observed.

## Abbreviations

ANOVA: Analysis of variance
CD1: Standard nomenclature for outbred mice
DMEM: Dulbecco’s modified Eagle’s medium
DHFR: Dihydrofolatereductase
LC-MS/MS: Liquid Chromatography Tandem Mass Spectrometry
MRM: Multiple reaction monitoring
NTDs: Neural tube defects
TMP-SMZ: Trimethoprim-sulfamethoxazole
