# Supplementary figures and images for: Cranial neural tube defect after trimethoprim exposure
Source: BMC Res Notes. 2018 Jul 16;11:475. doi: 10.1186/s13104-018-3593-1 (PMC6048906; doi:10.1186/s13104-018-3593-1)

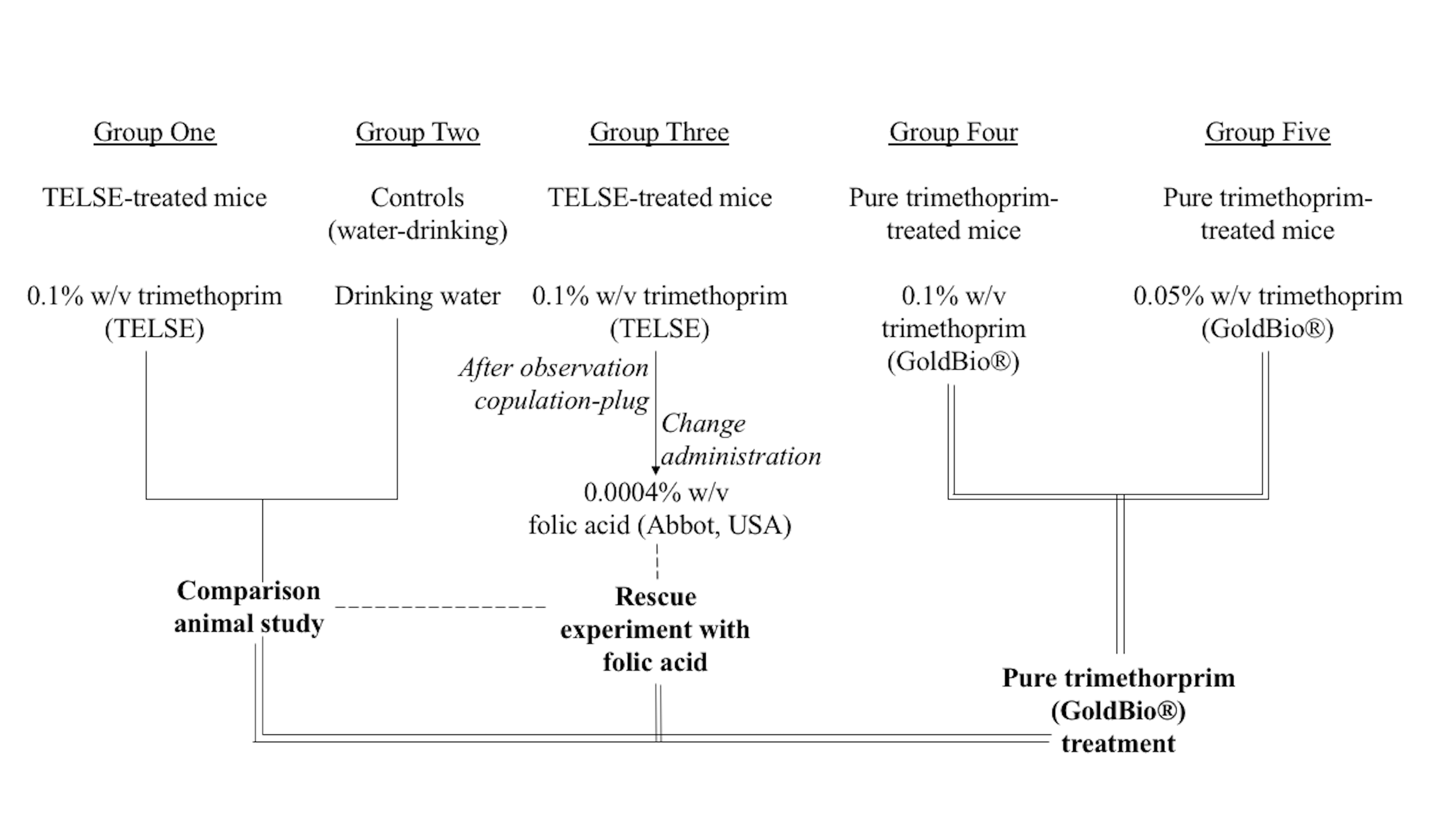

Supplement: Supplementary file 1 — Additional file 1. Schematic for animal study to look for effect of trimethoprim on primary neurulation E10.5. [file 13104_2018_3593_MOESM1_ESM.tiff]

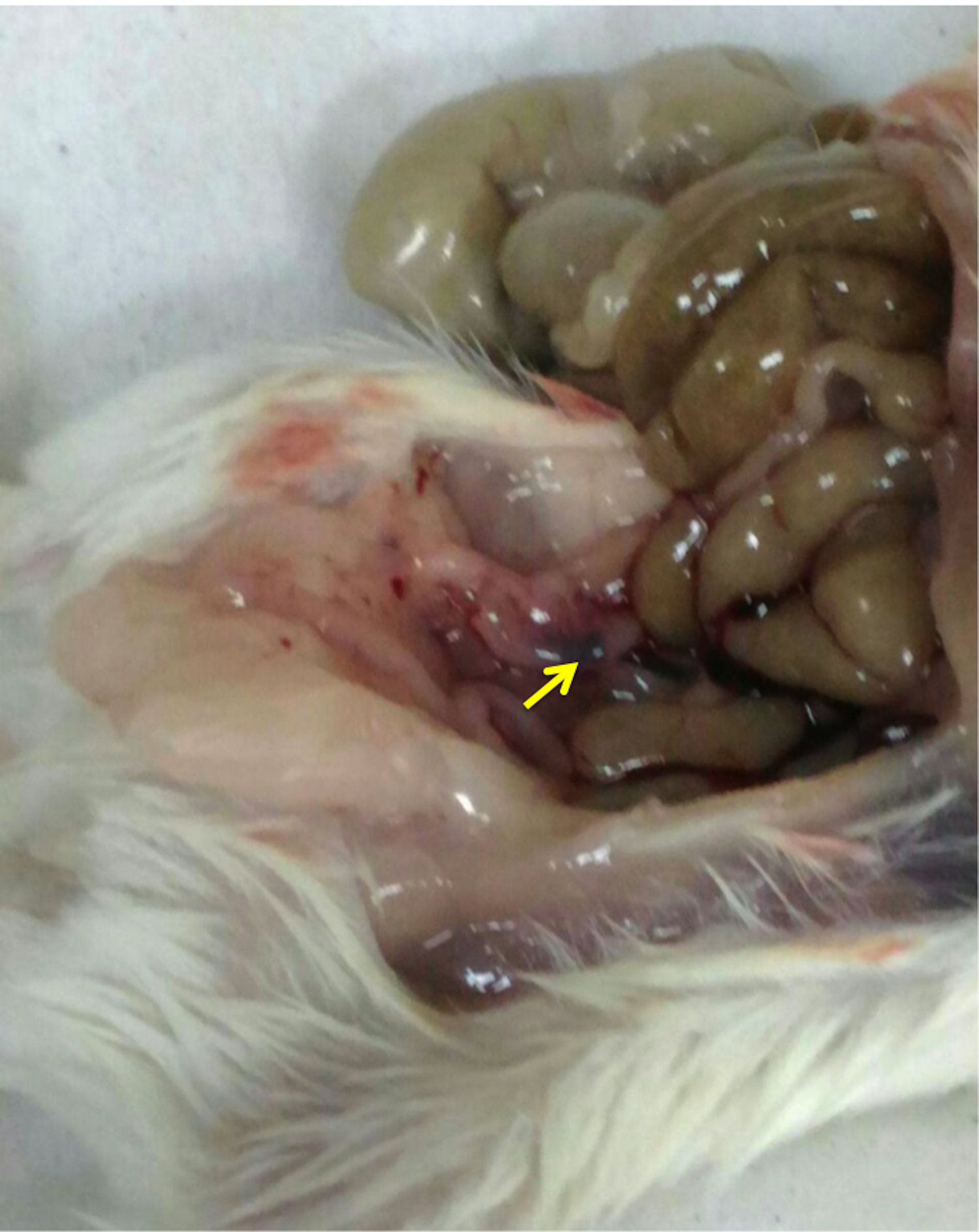

Supplement: Supplementary file 2 — Additional file 2. Unsuccessful pregnancy showing engorged mouse uterine horn. Despite repeated attempts at mating and with the presence of vaginal plugs, upon dissection, the engorged uterine horn with blood clots were instead observed. [file 13104_2018_3593_MOESM2_ESM.tiff]
